# Surgery Plays a Leading Role in Breast Cancer Treatment for Patients Aged ≥90 Years: A Large Retrospective Cohort Study

**DOI:** 10.1245/s10434-024-15790-z

**Published:** 2024-08-04

**Authors:** Massimo Ferrucci, Daniele Passeri, Francesco Milardi, Andrea Francavilla, Matteo Cagol, Mariacristina Toffanin, Giacomo Montagna, Alberto Marchet

**Affiliations:** 1 Breast Surgery Unit, Veneto Institute of Oncology IOV, Istituto di Ricovero e Cura a Carattere Scientifico (IRCCS), Padova, Italy; 2 General Surgery, Department of Surgery, Oncology and Gastroenterology, University of Padova, Padova, Italy; 3 Unit of Biostatistics, Epidemiology and Public Health, Department of Cardiothoracic Vascular Sciences and Public Health, University of Padova, Padova, Italy; 4 Breast Service, Department of Surgery, Memorial Sloan Kettering Cancer Center, New York, NY USA

**Keywords:** Breast cancer, Elderly patients, Breast surgery, Axillary surgery, Geriatric assessment, Nonagenarians

## Abstract

**Background:**

The population aged ≥90 years is increasing worldwide, yet nearly 50% of elderly breast cancer (BC) patients receive suboptimal treatments, resulting in high rates of BC-related mortality. We analyzed clinical and survival outcomes of nonagenarian BC patients to identify effective treatment strategies.

**Methods:**

This single-institution retrospective cohort study analyzed patients aged ≥90 years diagnosed with stage I–III BC between 2007 and 2018. Patients were categorized into three treatment groups: traditional surgery (TS), performed according to local guidelines; current-standard surgery (CS), defined as breast surgery without axillary surgery (in concordance with 2016 Choosing Wisely guidelines) and/or cavity shaving; and non-surgical treatment (NS). Clinicopathological features were recorded and recurrence rates and survival outcomes were analyzed.

**Results:**

We collected data from 113 nonagenarians with a median age of 93 years (range 90–99). Among these patients, 43/113 (38.1%) underwent TS, 34/113 (30.1%) underwent CS, and 36/113 (31.9%) underwent NS. The overall recurrence rate among surgical patients was 10.4%, while the disease progression rate in the NS group was 22.2%. Overall survival was significantly longer in surgical patients compared with NS patients (*p* = 0.04). BC-related mortality was significantly higher in the NS group than in the TS and CS groups (25.0% vs. 0% vs. 7.1%, respectively; *p* = 0.01*)*. There were no significant differences in overall survival and disease-free survival between the TS and CS groups (*p* = 0.6 and *p* = 0.8, respectively), although the TS group experienced a significantly higher overall postoperative complication rate (*p* < 0.001).

**Conclusions:**

Individualized treatment planning is essential for nonagenarian BC patients. Surgery, whenever feasible, remains the treatment of choice, with CS emerging as the best option for the majority of patients.

The proportion of the elderly population is increasing worldwide. In 2020, the population aged 90 years or older increased by 10% compared with 50 years earlier,^1^ with faster growth of people aged >80 years of age compared with those >65 years of age.^2^ Breast cancer (BC) incidence has also risen with age. In 2020, 45% of new BC cases were diagnosed in women aged over 65 years, with one in nine of these cases occurring in nonagenarians.^3^ Nearly 50% of elderly BC patients receive suboptimal or unconventional treatments,^4^ reflecting a lack of standardized therapeutic approaches,^5^ mainly due to under-enrollment in specific clinical trials.^6^ Despite pre-existing comorbidities and lower life expectancy,^7^ 40% of women aged ≥80 years with BC die from cancer-related causes,^8^ mainly attributed to less aggressive management, resulting in poorer disease-free survival (DFS) and overall survival (OS) rates.^9^ Surgery remains the gold-standard treatment for stage I and II BC in elderly patients.^10^ Both mastectomy and breast conserving surgery achieve superior local control compared with primary endocrine therapy;^11^ however, an appropriate management should consider patient frailty and background as integral parts of treatment planning.^12^ A comprehensive geriatric assessment is crucial to identify elderly patients who can tolerate treatments while balancing factors influencing clinical and survival outcomes.^13^ We evaluated the clinical and survival outcomes in patients aged ≥90 years newly diagnosed with BC, and to assess which therapeutic approach is associated with the best results.

## Methods

This single-center, retrospective cohort study was conducted at the Veneto Institute of Oncology. We included consecutive patients aged ≥90 years with newly diagnosed stage I–III BC treated between January 2007 and December 2018. Patients with stage IV disease were excluded. A minimum follow-up period of 5 years was guaranteed whenever the survival period allowed. This study adhered to the Strengthening the Reporting of Observational Studies in Epidemiology (STROBE) guidelines^14^ and the study protocol was approved by the local Ethics Committee (CESC-IOV 2023-12).

### Clinical Features and Geriatric Assessment

Complete clinical features were collected for all the patients from our institutional database.

All patients underwent comprehensive geriatric evaluation, which included the Karnofsky Performance Status (KPS) scale to evaluate functional abilities; the Eastern Cooperative Oncology Group Performance Status (ECOG PS) to measure functional status; the Age-not Charlson Comorbidity Index (AN-CCI) to categorize comorbidities and evaluate mortality risk (excluding age due to the uniformly aged very elderly population); the Mini-Mental State Examination (MMSE) for cognitive function assessment; the Geriatric Depression Scale (GDS) for psychological evaluation; and body mass index (BMI) to assess the nutritional status.

### Therapeutic Approaches

All therapeutic approaches were described (surgery, hormone therapy [HT], chemotherapy, and radiotherapy) and the following surgical aspects were examined: type of breast surgery (mastectomy vs. wide local excision [WLE]); axillary surgery (sentinel lymph node biopsy [SLNB] and/or axillary lymph node dissection [ALND]); margin status of the specimen (defined as positive if ‘ink on tumor’ was present, and close if any margin width was <2 mm); and postoperative complications. The TNM staging system was adopted^15^ and tumor subtypes were categorized according to the World Health Organization biomolecular classification.^16^ Within the medical therapies, we comprehensively documented the treatment pathway, timing, and the administered drugs. Any clinical response to medical therapies was recorded according to the Response Evaluation Criteria in Solid Tumors (RECIST) criteria.^17^ Radiotherapy treatment fields were specified.

### Treatment Groups

Patients were stratified into three distinct groups according to the treatment received: traditional surgery (TS), current-standard surgery (CS), and non-surgical treatment (NS). TS was defined as per local guidelines, mainly adopted before 2016,^18^ and involving either mastectomy or WLE with margin cavity shaving^19^ possibly combined with axillary surgery, such as SLNB and/or ALND. CS was defined as either mastectomy or WLE alone, without axillary surgery, even for staging purposes only (according to the 2016 Society of Surgical Oncology Choosing Wisely guidelines),^20^ or additional surgical procedures (e.g. margin cavity shaving).

### Study Endpoints and Statistical Analysis

The main endpoints were OS, DFS, progression-free survival (PFS) and recurrence rate (RR). DFS analysis was limited to surgical patients, while PFS analysis was exclusive to NS patients. Categorical variables are expressed as numbers (%) and compared using the Chi-square or Fisher's exact tests. Continuous variables are expressed as median ± interquartile range (IQR) and compared using the Wilcoxon or Kruskal–Wallis rank-sum test.

Given the advanced age of the cohort, the median follow-up time was estimated using the reverse Kaplan–Meier estimator,^21^ which combines Korn’s potential follow-up advantage to overcome the bias of underestimating the follow-up time.

OS, DFS, and PFS were evaluated using Kaplan–Meier survival analysis, employing the log-rank test to compare different patient groups. Cancer-specific survival (CSS) was assessed through competing risk analysis, considering deaths from non-BC causes as competing risks. The Gray test was used to compare the surgical and non-surgical groups.

A multiple Cox proportional hazards regression model was built to assess whether any differences in OS could be attributed to the type of surgery (TS vs. CS). To partially overcome the limitations of retrospective studies and address potential sources of bias, this model was adjusted for well-established BC prognostic factors. This was achieved by considering the number of events that occurred and trying to mitigate any collinearity between our predictors, i.e., age, type of surgery, Charlson score, and tumor stage. The results are expressed as hazard ratios (HR) with 95% confidence intervals (CI) and associated *p*-values. A *p*-value <0.05 was considered statistically significant. Statistical analyses were performed using R software.^22^

## Results

This study included 113 nonagenarian Caucasian patients living in Italy, of whom only one patient was male. Of these patients, 43 (38.1%) underwent TS, 34 (30.1%) underwent CS, and 36 (31.9%) did not undergo surgery (and represent the NS group).

### Geriatric Assessment

The median age of the entire cohort was 93 years (range 90–99). The most common comorbidities were hypertension (66.4%), osteoporosis (29.2%), diabetes (24.8%), and atrial fibrillation (23.0%) [Table 1]. Approximately half of the patients presented with a good clinical condition, as indicated by Karnofsky scores of 80–100 (54.9%), ECOG scores of 0–1 (54.9%), and AN-CCI scores of 0–1 (48.6%). Cognitive function was normal in 54.9% of patients, and 74.3% exhibited normal humoral status according to the GDS.

**Table 1:** Patients' and tumors’ characteristics

Patients' variables	Overall population [*N* = 113]	Traditional Surgery [*n* = 43] (38.1%)	Current standard Surgery [*n* = 34] (30.1%)	No Surgery [*n* = 36] (31.9%)	*p*-value^a^
Patients’ clinical characteristics [n (%)]					
Age (years)					0.020
Median (IQR)	93 (91–94)	91 (90–93)	92 (91–94)	93 (91–94)	
Range	90–99	90–99	90–96	90–96	
Sex					0.300
Male	1 (0.9)	0 (0.0)	1 (2.9)	0 (0.0)	
Female	112 (99.1)	43 (100)	33 (97.1)	36 (100)	
Karnofsky PS scale					0.090
80–100	62 (54.9)	27 (62.8)	21 (61.8)	14 (38.9)	
50–70	49 (43.4)	16 (37.2)	12 (35.3)	21 (58.3)	
10–40	2 (1.8)	0 (0.0)	1 (2.9)	1 (2.8)	
ECOG PS score					0.010
0	19 (16.8)	7 (16.3)	10 (29.4)	2 (5.6)	
1	43 (38.1)	21 (48.8)	12 (35.3)	10 (27.8)	
2	35 (31.0)	13 (30.2)	8 (23.5)	14 (38.9)	
3	14 (12.4)	2 (4.7)	3 (8.8)	9 (25.0)	
4	2 (1.8)	0 (0.0)	1 (2.9)	1 (2.8)	
Age-not Charlson Comorbidity Index					0.300
0	24 (21.2)	7 (16.3)	9 (26.5)	8 (22.2)	
1	31 (27.4)	15 (34.9)	10 (29.4)	6 (16.7)	
2 or 3	46 (40.7)	19 (44.2)	11 (32.4)	16 (44.4)	
≥4	12 (10.6)	2 (4.7)	4 (11.8)	6 (16.7)	
Mini-Mental State Examination					0.020
Normal cognitive function	62 (54.9)	27 (62.8)	20 (58.8)	15 (41.7)	
Borderline cognitive decline	12 (10.6)	9 (20.9)	1 (2.9)	2 (5.6)	
Mild cognitive decline	36 (31.9)	7 (16.3)	13 (38.2)	16 (44.4)	
Serious cognitive decline	3 (2.7)	0 (0.0)	0 (0.0)	3 (8.3)	
Geriatric Depression Scale					<0.001
Normal humoral status	84 (74.3)	37 (86.0)	26 (76.5)	21 (58.3)	
Mild depressive status	26 (23.0)	6 (14.0)	5 (14.7)	15 (41.7)	
Serious depressive status	3 (2.7)	0 (0.0)	3 (8.8)	0 (0.0)	
Body mass index					0.350
<18.5	4 (3.5)	1 (2.3)	2 (5.9)	1 (2.8)	
18.5–24.9	38 (33.6)	14 (32.6)	11 (32.4)	13 (36.1)	
25.0–29.9	50 (44.2)	24 (55.8)	13 (38.2)	13 (36.1)	
≥30	21 (18.6)	4 (9.3)	8 (23.5)	9 (25.0)	
Comorbidities					<0.001
Hypertension	75 (66.4)	29 (67.4)	21 (61.8)	25 (69.4)	
Osteoporosis	33 (29.2)	15 (34.9)	8 (23.5)	10 (27.8)	
Diabetes	28 (24.8)	9 (20.9)	5 (14.7)	14 (38.9)	
Atrial fibrillation	26 (23.0)	6 (14.0)	7 (20.6)	13 (36.1)	
COPD	12 (10.6)	6 (14.0)	2 (5.9)	4 (11.1)	
Renal failure	9 (8.0)	1 (2.3)	4 (11.8)	4 (11.1)	
BC family history					0.700
Yes	20 (17.7)	7 (16.3)	6 (17.6)	7 (19.4)	
No	93 (82.3)	36 (83.7)	28 (82.4)	29 (80.6)	
Previous neoplasm					0.300
Ipsilateral breast	9 (8.0)	5 (11.6)	2 (5.9)	2 (5.6)	
Controlateral breast	12 (10.6)	5 (11.6)	4 (11.8)	3 (8.3)	
Other sites	10 (8.8)	3 (7.0)	4 (11.8)	3 (8.3)	
Diagnostic imaging assessment [n (%)]					
Ultrasound	113 (100)	43 (100)	34 (100)	36 (100)	
Mammography	113 (100)	43 (100)	34 (100)	36 (100)	
Magnetic resonance	8 (7.1)	5 (11.6)	3 (8.8)	0 (0.0)	
Lump biopsy	113 (100)	43 (100)	34 (100)	36 (100)	
Lymph node biopsy	15 (13.3)	6 (14.0)	1 (2.9)	8 (22.2)	
Systemic staging assessment [n (%)]					
Staged cases	71 (62.8)	30 (69.7)	19 (55.9)	22 (61.1)	

Tumors' variables	Overall cases [*N* = 118]	Traditional Surgery [*n* = 44] (37.6%)	Current standard Surgery [*n* = 35] (30.0%)	No Surgery [*n* = 39] (32.5%)	*p*-value^a^
Tumor macroscopic characteristics [n (%)]					
Laterality^b^					0.510
Unilateral	108 (95.6)	42 (97.7)	33 (97.1)	33 (91.7)	
Bilateral	5 (4.4)	1 (2.3)	1 (2.9)	3 (8.3)	
Distribution					0.810
Unifocal	112 (94.9)	40 (90.9)	33 (94.3)	39 (100)	
Multifocal	4 (3.4)	2 (4.5)	2 (5.7)	0 (0.0)	
Multicentric	2 (1.7)	2 (4.5)	0 (0.0)	0 (0.0)	
Palpable mass					0.780
Yes	115 (97.5)	42 (95.5)	35 (100)	38 (97.4)	
No	3 (2.5)	2 (4.5)	0 (0.0)	1 (2.6)	
Palpable axillary nodes					0.002
Yes	26 (22.0)	9 (20.5)	2 (5.7)	15 (38.5)	
No	92 (78.0)	35 (79.5)	33 (94.3)	24 (61.5)	
Visible morphological features					0.200
No	84 (71.2)	34 (77.3)	28 (80.0)	22 (56.4)	
Skin ulceration	16 (13.6)	5 (11.4)	5 (14.3)	6 (15.4)	
Nipple alterations	7 (5.9)	2 (4.5)	1 (2.9)	4 (10.3)	
Other	11 (9.3)	3 (6.8)	1 (2.9)	7 (17.9)	
Tumor histologic characteristics [n (%)]					
Histotype					0.370
IC NST	93 (78.8)	33 (75.0)	28 (80.0)	32 (82.1)	
ILC	23 (19.5)	11 (25.0)	5 (14.3)	7 (17.9)	
DCIS	0 (0.0)	0 (0.0)	0 (0.0)	0 (0.0)	
Other	2 (1.7)	0 (0.0)	2 (5.7)	0 (0.0)	
Biomolecular classification					0.050
Luminal A	100 (84.7)	38 (86.4)	27 (77.1)	35 (89.7)	
Luminal B	7 (5.9)	0 (0.0)	4 (11.4)	3 (7.7)	
HER2+	11 (9.3)	6 (13.6)	4 (11.4)	1 (2.6)	
Triple negative	0 (0.0)	0 (0.0)	0 (0.0)	0 (0.0)	
Grading					0.050
Well differentiated	12 (10.2)	6 (13.6)	6 (17.1)	0 (0.0)	
Moderately differentiated	62 (52.5)	21 (47.7)	15 (42.9)	26 (66.7)	
Poorly differentiated	44 (37.3)	17 (38.6)	14 (40.0)	13 (33.3)	
Main pathological dimension (mm)					
Median (IQR)	27 (18–36)	28 (18–40)	21 (14–27)	31 (27–35)	<0.001
Staging [n (%)]					
T-TNM					<0.001
T1	35 (29.7)	13 (29.5)	19 (54.3)	3 (7.7)	
T2	59 (50.0)	22 (50.0)	10 (28.6)	27 (69.2)	
T3	2 (1.7)	2 (4.5)	0 (0.0)	0 (0.0)	
T4	22 (18.6)	7 (15.9)	6 (17.1)	9 (23.1)	
N-TNM					<0.001
N0	92 (78.0)	30 (68.2)	35 (100)	27 (69.2)	
N1	22 (18.6)	11 (25.0)	0 (0.0)	11 (28.2)	
N2	4 (3.4)	3 (6.8)	0 (0.0)	1 (2.6)	
Stage					<0.001
I	21 (17.8)	5 (11.4)	15 (42.9)	1 (2.6)	
II	73 (61.9)	29 (65.9)	15 (42.9)	29 (74.4)	
III	24 (20.3)	10 (22.7)	5 (14.3)	9 (23.1)	

*PS* performance status, *ECOG* Eastern Cooperative Oncology Group, *COPD* chronic obstructive pulmonary disease, *BC* breast cancer, *IC NST* invasive carcinoma of no-special type, *ILC* invasive lobular carcinoma, *DCIS* ductal carcinoma in situ, *HER2+* human epithelial growth factor receptor 2-positive, *IQR* interquartile range

All percentages have been rounded to one decimal place

^a^Fisher's exact test, Pearson’s Chi-square test, Wilcoxon and Kruskal–Wallis rank-sum tests

^b^Referred to 113 overall patients (43 in the TS cohort, 34 in the CS cohort, 36 in the NS cohort)

### Neoplastic Features and Staging

Five patients presented with bilateral tumors. A palpable mass was the clinical presentation in 115/118 cases (97.5%), with 28.8% also showing visible findings and 22.0% showing palpable axillary nodes. Most tumors were invasive carcinomas of no special type (78.8%) and were categorized as Luminal A (84.7%). Neither ductal carcinoma in situ nor triple-negative BC cases were recorded. The global median tumor size was 27 mm, with significant differences among the groups (28 mm in the TS group, 21 mm in the AS group, and 31 mm in the NS group; *p* < 0.001). At presentation, half of the cases (50.0%) were classified as T2 neoplasia, while T4 accounted for 18.6% of cases. Lymph node metastases were detected in 22.0% of cases (Table 1).

### Primary Systemic Therapy

Primary systemic therapy (PST), always hormone-based, was administered to 48/113 patients (42.5%), of whom 12/48 (25.0%) underwent subsequent surgery, while 36 (31.9% of the entire cohort) did not (Table 2).

**Table 2:** Therapeutic approaches and clinical outcomes

	Overall cases	Traditional Surgery	Current standard Surgery	No Surgery	*p*-value^a^
Neoadjuvant therapies [n/N (%)]					
Neoadjuvant HT	**12/77 (15.6)**	**8/43 (18.6)**	**4/34 (11.8)**	**0/36 (0.0)**	<0.001
Median duration (IQR), months	19 (9–32)	19 (10–25)	9 (7–12)		
Drug					<0.001
Tamoxifen	1/12 (8.3)	1/8 (12.5)	0/4 (0.0)		
Anastrozole	3/12 (25.0)	3/8 (37.5)	0/4 (0.0)		
Letrozole	8/12 (66.7)	4/8 (50.0)	4/4 (100)		
Response to therapy^b^					0.400
Complete response	0/12 (0.0)	0/8 (0.0)	0/4 (0.0)		
Partial response	8/12 (66.7)	5/8 (62.5)	3/4 (75.0)		
Stable disease	0/12 (0.0)	0/8 (0.0)	0/4 (0.0)		
Progression disease	4/12 (33.3)	3/8 (37.5)	1/4 (25.0)		
Surgical treatments [n/N (%)]					
Surgical patients	77/113 (68.1)	43/43 (100)	34/34 (100)	0/36 (0.0)	
Upfront surgery	65/77 (84.4)	35/43 (81.4)	30/34 (88.2)		
Breast surgery					<0.001
Mastectomy	31/79 (39.2)	27/44 (61.4)	4/35 (11.4)		
WLE	48/79 (60.8)	17/44 (38.6)	31/35 (88.6)		
Axillary surgery for cN0 patients					
cN0 patients	65/77 (84.4)	31/43 (72.1)	34/34 (100)		
SLNB	30/79 (38.0)	30/44 (68.2)	0/35 (0.0)		
ALND	0/79 (0.0)	0/44 (0.0)	0/35 (0.0)		
SLNB + ALND	2/79 (2.5)	2/44 (4.5)	0/35 (0.0)		
Axillary surgery for cN+ patients					
cN+ patients	12/77 (15.6)	12/43 (27.9)	0/34 (0.0)		
SLNB	0/79 (0.0)	0/44 (0.0)	0/35 (0.0)		
ALND	12/79 (15.2)	12/44 (27.3)	0/35 (0.0)		
SLNB + ALND	0/79 (0.0)	0/44 (0.0)	0/35 (0.0)		
Anesthesia					<0.010
General	37/77 (48.1)	34/43 (79.0)	3/34 (8.8)		
Local with sedation	35/77 (45.5)	6/43 (14.0)	29/34 (85.3)		
Locoregional block	5/77 (6.5)	3/43 (7.0)	2/34 (5.9)		
Margins					0.300
Close	11/79 (13.9)	5/44 (11.4)	6/35 (17.1)		
Positive	9/79 (11.4)	3/44 (6.8)	6/35 (17.1)	.	
Negative	59/79 (74.7)	36/44 (81.8)	23/35 (65.7)		
Recurrences or progression disease^c^ [n/N (%)]	*Vital status [n/N (%)]*				
Total of recurrences/progression disease	16/113 (14.2)	5/43 (11.6)	3/34 (8.8)	8/36 (22.2)	0.049
Type of recurrence/progression disease					
Local	7/16 (43.8)	3/5 (60.0)	2/3 (66.7)	2/8 (25.0)	
Locoregional	4/16 (25.0)	1/5 (20.0)	1/3 (33.3)	2/8 (25.0)	
Distant	3/16 (18.8)	0/5 (0.0)	0/3 (0.0)	3/8 (37.5)	
Local + distant	2/16 (12.5)	1/5 (20.0)	0/3 (0.0)	1/8 (12.5)	
Recurrence/PD treatment					
Surgery	3/16 (18.8)	3/5 (60.0)	0/3 (0.0)	0/8 (0.0)	
Hormone therapy	9/16 (56.3)	4/5 (80.0)	1/3 (33.3)	4/8 (50.0)	
Chemotherapy	1/16 (6.3)	0/5 (0.0)	0/3 (0.0)	1/8 (12.5)	
Palliative care	5/16 (31.3)	0/5 (0.0)	2/3 (66.7)	3/8 (37.5)	

	Overall cases	Traditional Surgery	Current standard Surgery	No Ssurgery	*p*-value^a^
Margin revision					<0.001
Yes	3/20 (15.0)	3/8 (37.5)	0/12 (0.0)		
No	17/20 (85.0)	5/8 (62.5)	12/12 (100)		
Complications					<0.001
Patients with at least one complication	40/77 (51.9)	32/43 (74.4)	8/34 (23.5)		
Breast seroma	18/79 (22.8)	12/44 (27.3)	6/35 (17.1)		
Lymphocele	14/79 (17.7)	14/44 (31.8)	0/35 (0.0)		
Lymphedema	8/79 (10.1)	8/44 (18.2)	0/35 (0.0)		
Arm numbness	4/79 (5.1)	4/44 (9.1)	0/35 (0.0)		
Wound dehiscence	3/79 (3.8)	1/44 (2.3)	2/35 (5.7)		
Infection	3/79 (3.8)	3/44 (6.8)	0/35 (0.0)		
Adjuvant therapies					
Adjuvant hormone therapy	56/77 (72.7)	32/43 (74.4)	24/34 (70.6)	0/36 (0.0)	<0.001
Median duration (IQR), months	34 (18–61)	37 (25–61)	33 (15–49)		
Drug					
Tamoxifen	5/56 (8.9)	1/32 (3.1)	4/24 (16.7)		
Anastrozole	9/56 (16.1)	6/32 (18.8)	3/24 (12.5)		
Letrozole	29/56 (51.8)	18/32 (56.3)	11/24 (45.8)		
Exemestane	13/56 (23.2)	7/32 (21.9)	6/24 (25.0)		
Suspended for AE	4/56 (7.1)	0/32 (0.0)	4/24 (16.7)		
Adjuvant radiotherapy	7/77 (9.1)	6/43 (14.0)	1/34 (2.9)		0.010
Indicated but not administered	14/77 (18.2)	4/43 (9.3)	10/34 (29.4)		
Breast RT after WLE	4/7 (57.1)	3/6 (50.0)	1/1 (100)		
Chest wall RT	1/7 (14.3)	1/6 (16.7)	0/1 (0.0)		
Regional LN RT	2/7 (28.6)	2/6 (33.3)	0/1 (0.0)		
Non-surgical therapies as definitive treatment [n/N (%)]					
Hormone therapy	36/113 (31.9)	0/43 (0.0)	0/34 (0.0)	36/36 (100)	
Median duration (IQR), months	24 (11-33)			24 (11-33)	
Drug					
Tamoxifen	5/36 (13.9)			5/36 (13.9)	
Anastrozole	2/36 (5.6)			2/36 (5.6)	
Letrozole	25/36 (69.4)			25/36 (69.4)	
Exemestane	4/36 (11.1)			4/36 (11.1)	
Response to therapy^b^					
Complete response	2/36 (5.6)			2/36 (5.6)	
Partial response	24/36 (66.7)			24/36 (66.7)	
Stable disease	2/36 (5.6)			2/36 (5.6)	
Progression disease	8/36 (22.2)			8/36 (22.2)	
Vital status [n/N (%)]					
Alive	51/113 (45.1)	23/43 (53.5)	20/34 (58.8)	8/36 (22.2)	<0.001
Dead	62/113 (54.9)	20/43 (46.5)	14/34 (41.2)	28/36 (77.8)	
Deaths due to breast cancer	8/62 (12.9)	0/20 (0.0)	1/14 (7.1)	7/28 (25.0)	0.010
Deaths not due to breast cancer	54/62 (87.1)	20/20 (100)	13/14 (92.9)	21/28 (75.0)	
Heart failure	27/62 (43.5)	12/20 (60.0)	8/14 (57.1)	7/28 (25.0)	
Respiratory failure	12/62 (19.4)	5/20 (25.0)	1/14 (7.1)	6/28 (21.4)	
Other cancers	8/62 (12.9)	1/20 (5.0)	2/14 (14.3)	5/28 (17.9)	
Stroke	4/62 (6.5)	1/20 (5.0)	0/14 (0.0)	3/28 (10.7)	
Renal failure	2/62 (3.2)	1/20 (5.0)	1/14 (7.1)	0/28 (0.0)	
Bowel obstruction	1/62 (1.6)	0/20 (0.0)	1/14 (7.1)	0/28 (0.0)	

*HT* hormone therapy, *WLE* wide local excision, *SLNB* sentinel lymph node biopsy, *ALND* axillary lymph node dissection, *AE* adverse effects, *RT* radiotherapy, *LN* lymph node, *PD* progression disease, *IQR* interquartile range, *RECIST* Response Evaluation Criteria in Solid Tumors

All percentages have been rounded to one decimal place

^a^Fisher's exact test, Pearson’s Chi-square test, Wilcoxon and Kruskal–Wallis rank-sum tests

^b^Treatment responses were assessed according to the RECIST criteria^17^

^c^Recurrences are reported for surgical patients, progression disease is reported for non-surgical patients

HT as a definitive treatment was delivered to 31.9% of patients, with a median duration of 24 months, predominantly using aromatase inhibitors (AIs; 86.1%). Among these patients, 30.6% had to switch type of HT due to adverse effects, most commonly arthralgia. Partial clinical response was documented in 66.7% of patients, while two patients achieved complete clinical response; disease progression was recorded in 22.2% of patients, while two patients showed stable disease. Neoadjuvant HT was administered to 15.6% of the surgical patients, mainly using AIs (91.7%), for a median duration of 19 months. This approach was chosen for patients who were initially non-surgical candidates due to transient comorbidities, those who needed a reduction in BC dimensions to become operable, or for patients who initially refused surgery. Of these patients, 66.7% showed partial response, whereas 33.3% experienced disease progression. The following surgical treatment was TS and CS for 8 and 4 of these patients, respectively.

Chemotherapy was not administered to any patients, neither in the neoadjuvant setting nor as part of an exclusive medical treatment regimen. This decision was mainly due to the frailty of this specific age group of patients. After multidisciplinary discussion, it was determined that the risks outweighed the potential benefits, even in cases of human epidermal growth factor receptor 2-positive tumors, where trastuzumab would have posed a risk of severe cardiotoxicity, especially in patients already suffering from heart disease.

### Surgical Treatment

Seventy-seven patients (68.1% of the entire cohort, corresponding to 79 tumors) underwent surgery, of whom 65 (84.4%) underwent upfront surgery. Mastectomy was performed in 39.2% of tumors, while 60.8% underwent WLE. Mastectomy was the most common procedure in the TS group (27/44, 61.4%), while WLE was preferred in the CS group (31/35, 88.6%; *p* < 0.001). Axillary surgery was only performed in the TS group, with SLNB performed in 30/44 cN0 cases (68.2%) and ALND in all 12 cN+ patients. In two cases, sentinel lymph nodes were positive and ALND was consequently performed.

The overall positive and close margin rates were 11.4% and 13.9%, respectively, and no significant differences were observed between the two surgical groups (*p* = 0.151). Re-operation for margin clearance was performed in three TS selected cases with positive margins, while no close margin was considered for additional surgery.

The overall rate of patients with at least one postoperative complication was 51.9%, with breast seroma being the most frequent (22.8% of all procedures), occurring after 41.9% of mastectomies and 10.4% of WLEs (*p* < 0.001). The TS group experienced a significantly higher postoperative complication rate, including minor complications, than the CS group (74.4% vs. 23.5%; *p* < 0.001), with a reported axillary lymphocele rate of 31.8%, mainly after ALND (71.4% vs. 13.3% after SLNB; *p* < 0.01). Mild arm lymphedema was recorded after ALND in 18.2% of TS procedures. All postoperative complications were conservatively managed and no surgical re-intervention was required. The 30-day postoperative mortality rate was zero.

### Adjuvant Therapies

Adjuvant HT was administered to 72.7% of surgical patients, with a median duration of 34 months; in 7.1% of cases, adjuvant HT was suspended due to adverse effects. AIs were used in 91.1% of patients. None of the patients received adjuvant chemotherapy. Adjuvant radiotherapy was recommended for 21/77 surgical patients (27.3%), but only 9.1% adhered to the treatment.

### Recurrences and Disease Progression

Among all surgical patients, the RR was 10.4%, whereas the NS group experienced an overall (local and/or distant) disease progression rate of 22.2% (*p* = 0.049*).* Recurrence management consisted of additional surgery in the TS group only, performed in three of five recorded recurrences (60.0%). Specifically, this included two WLEs in patients initially staged IIB who subsequently developed ipsilateral BC recurrence, and one ALND in a stage IIIB patient with axillary recurrence.

HT was the treatment used for 80% of TS group recurrences and 33.3% of CS group recurrences. Additionally, 50% of NS patients experiencing disease progression underwent drug switching during the course of HT. Supportive care as the sole recurrence treatment was provided to 66.7% and 37.5% of CS and NS patients, respectively.

### Survival Outcomes

Over a median estimated follow-up of 77 months (real median follow-up of 42 months and a loss to follow-up rate of 7.4%), 54.9% (62/113) of the cohort died, with only 12.9% of these deaths caused by BC. BC-related mortality was significantly higher in the NS group than in the TS and CS groups (25% vs. 0% and 7.1%, respectively; *p* = 0.01*)*. The leading causes of non-BC-related deaths were heart failure (43.5%), respiratory failure (19.4%), and cancer at other sites (12.9%).

**Fig. 1:**
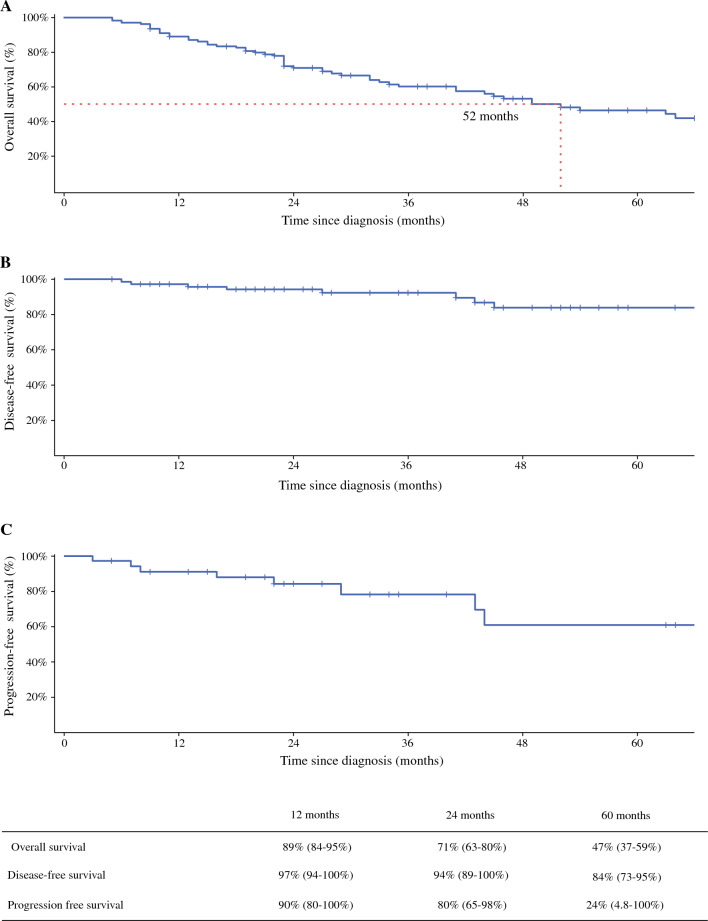
**a** Overall survival in the whole population; **b** disease-free survival in surgical patients; and **c** progression-free survival in non-surgical patients

**Fig. 2:**
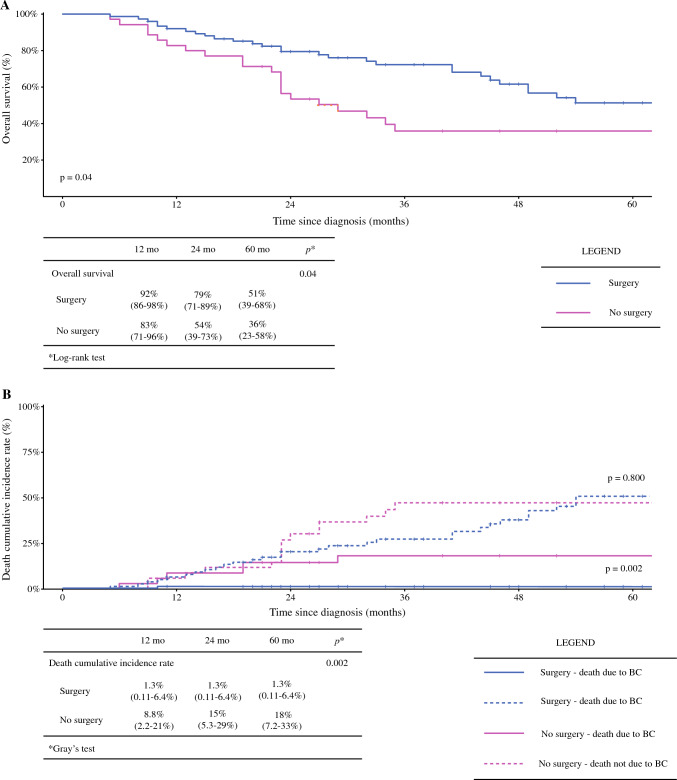
**a** Overall survival and **b** cumulative incidence of death in surgical versus non-surgical patients. *mo* months, *BC* breast cancer

**Fig. 3:**
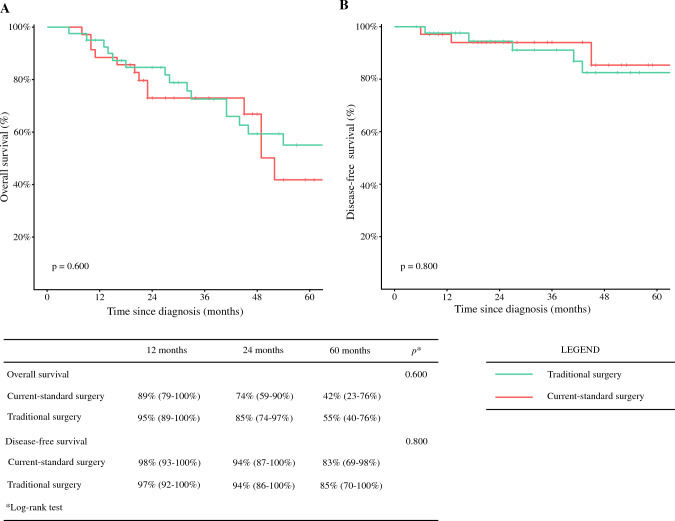
**a** Overall survival and **b** disease-free survival in all surgical patients: traditional surgery versus current-standard surgery

**Fig. 4:**
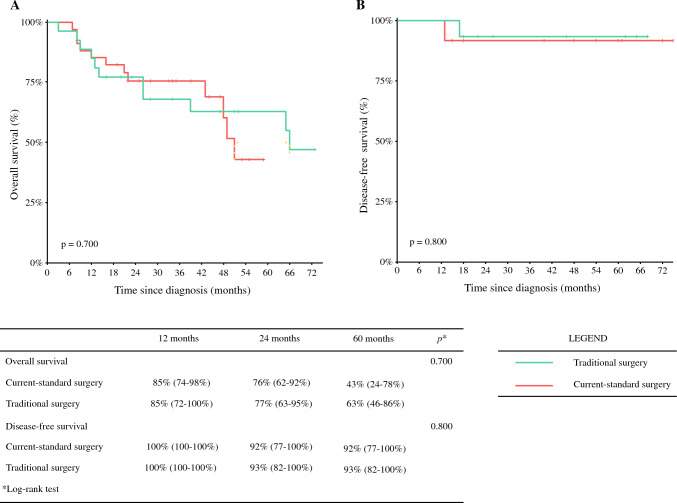
**a** Overall survival and **b** disease-free survival in cN0 surgical patients: traditional surgery versus current-standard surgery

The median global OS was 52 months, with median DFS and median PFS not reached in any of the three groups (Fig. 1). Surgical patients experienced a significantly higher OS than NS patients (*p* = 0.04) (Fig. 2a). When considering non-BC-related deaths as a competing risk, surgical patients showed a significantly lower BC-related mortality rate (*p* = 0.002), with no notable difference in mortality from other causes between the groups (*p* = 0.8) (Fig. 2b). No significant differences in OS or DFS were observed between the TS and CS groups (*p* = 0.6 and *p* = 0.8, respectively) (Fig. 3). A specific survival analysis revealed no significant difference in OS (medians of 66 and 51 months, respectively) and DFS between clinically node-negative (cN0) patients at diagnosis undergoing TS or CS (*p* = 0.7 and *p* = 0.8, respectively) (Fig. 4).

A Cox Model analysis, including all the surgical patients, revealed that the Charlson score at diagnosis was the only significant predictor of poorer OS (HR 1.24, 95% CI 1.03–1.48; *p* = 0.022). Conversely, age, tumor stage, and, most notably, the type of surgery did not significantly affect the OS (Table 3).

**Table 3:** Overall survival Cox proportional hazards regression model for surgical patients

	HR	95% CI	*p*-value
Type of surgery			
Current-standard Surgery	–	–	
Traditional Surgery	0.88	0.41–1.88	0.700
Age	1.00	0.82–1.23	>0.900
Charlson Comorbidity Index	1.24	1.03–1.48	0.022
Tumor stage			
I	–	–	
II	1.15	0.45–2.93	0.800
III	1.37	0.48–3.93	0.600

*HR* hazard ratio, *CI* confidence interval

## Discussion

The very elderly population remains significantly underrepresented in BC clinical trials due to challenges in their enrollment in prospective studies. This has led to a lack of evidence and limited recommendations for their optimal clinical management.^5^ Despite the increasing incidence of BC with age, older patients frequently face underdiagnosis, understaging, and undertreatment compared with their younger counterparts, resulting in increased mortality rates.^23,24^ To the best of our knowledge, this study represents the largest single-center analysis of BC treatment among nonagenarians to date. Similar to prior studies,^23^ almost all BC cases in our cohort (97.5%) were palpable. This highlights the impact of screening omission in elderly women, consistently with specific guidelines^25,26^ for this age group, resulting in delayed diagnosis.

### Comprehensive Geriatric Assessment

In the United States, individuals aged ≥ 90 years have a life expectancy of over 4.5 years,^27^ while in Europe, women aged 92–93 years have an overall 11.4% chance of reaching 100.^28^ Despite the advanced age at diagnosis, nearly half of our patients were in good clinical condition, making them potential candidates for surgery. Among these fragile patients, it is crucial to identify those who can tolerate the treatments, achieving survival benefits. Consequently, we conducted a comprehensive geriatric assessment for all elderly patients to predict adverse events and to determine the most suitable treatment plans. Our analysis identified the AN-CCI as the sole prognostic factor for OS within the surgical cohort, underlining its utility to evaluate a patient’s suitability for surgery.

### Benefits of Surgery

Rao et al. demonstrated that BC surgery offered survival advantages over primary HT in patients aged ≥ 80 years, with a significant decrease in local RR and improved 5-year survival rates.^29^ Similarly, we observed a lower RR (10.4% vs. 22.2%) and a better 5-year OS (51% vs. 36%) in surgical patients compared with the NS group. Di Lascio et al. retrospectively analyzed 58 BC patients aged ≥ 89 years and found that among surgical patients, 56% underwent mastectomy and 71% had axillary surgery, resulting in a 10% relapse rate and a median survival of 50 months.^30^ Our experience showed a median OS of 52 months, with lower axillary surgery (54.4%) and mastectomy (39.2%) rates; however, the mastectomy rate was significantly higher than that observed in the younger population with BC.^31^ This is likely due to a higher clinical T stage, contraindications to radiation therapy, and the omission of level II oncoplastic techniques in this demographic.

### Axillary Surgery

Elderly BC patients are less likely to undergo axillary surgery due to the lack of long-term survival benefit^32^ and increased risk of specific morbidities.^33^

Recently, the SOUND trial,^34^ along with other randomized trials, investigated SLNB omission in cN0 younger patients,^35^ suggesting that its implementation in the elderly is even more warranted. In our cohort, axillary surgery was performed in only 36.6% of patients, with no OS advantage for cN0 patients, supporting its omission. The International Breast Cancer Study Group demonstrated that avoiding axillary surgery in women aged ≥60 years diagnosed with cN0 BC improved their quality of life without adversely affecting DFS or OS.^36^

A 2016 consensus statement from the Society of Surgical Oncology^20^ declared SNLB avoidable in clinically node-negative women aged ≥70 years with early-stage hormone receptor-positive, HER2-negative invasive BC; however, our experience shows that current clinical practice still diverges from these recommendations.^37^ In our population, 49.2% of cN0 patients underwent SLNB (the majority were performed before 2016), while all 12 cN+ patients underwent ALND. A recent study involving 125 patients aged ≥65 years with favorable BC undergoing breast-conserving surgery without SLNB reported only 1.6% axillary recurrences, further supporting this approach.^38^

Future studies could explore the feasibility of totally omitting axillary surgery in nonagenarians even with involved axillary lymph nodes, relying on systemic therapy only. This strategy could reduce post-surgical complications without negatively affecting OS. Alternatively, only the excision of macroscopically involved lymph nodes in patients, or tailored axillary dissection, may be considered, as already being investigated by the TAXIS trial,^39^ and hopefully by further prospective studies.

### Radiotherapy

Adjuvant radiotherapy slightly reduces the risk of local recurrence, but does not significantly improve OS in older populations.^40–42^ It is crucial to balance its benefits against potential adverse effects, particularly among elderly patients.^43^ The St. Gallen International Consensus Guidelines recommend adjuvant radiotherapy for older women with a life expectancy exceeding 10 years.^44^ Consequently, the omission of radiotherapy for nonagenarians is justifiable because of their shorter life expectancies and the high incidence of contraindications due to their comorbidities. In our experience, radiotherapy was advised in selected patients (27.3%), although adherence was notably low (9.1%).

### Hormone Therapy

Fennessy et al. reported reduced OS and increased BC-related mortality in patients aged over 70 years treated with tamoxifen alone, compared with those receiving surgery plus tamoxifen. Significant differences emerged after 5 years, suggesting tamoxifen as a potential option for elderly estrogen receptor-positive BC patients with a life expectancy of <5 years.^45^ The Italian GRETA trial showed increased local disease progression in the tamoxifen-alone arm compared with the tamoxifen plus surgery arm, without differences in OS or BC-specific survival.^46^ In our cohort, 31.9% of patients received HT alone, experiencing significantly higher global relapse and BC-related mortality rates than surgical patients (22.2% vs. 10.4%, and 25.0% vs. 1.33%, respectively). A specific survival analysis revealed significantly improved OS in surgical patients compared with that in the NS group, even when considering only BC-related deaths.

### Chemotherapy

In our experience, chemotherapy was never administered to nonagenarian BC patients due to their frailty and comorbidities. Elkin et al. demonstrated a reduction in all-cause mortality by approximately 16% among older patients with estrogen receptor-negative BC who received systemic chemotherapy;^47^ however, older patients may have an increased risk of cardiac toxicity, treatment-induced myelodysplasia, or acute leukemia.^48,49^ Consequently, the indications for chemotherapy in patients aged ≥90 years remain very rare.

### Study Limitations

This study has several limitations. First, its retrospective, single-institution design may limit the generalizability of the findings. Despite representing the largest cohort within this specific age group reported in the literature, the sample size is relatively small. Additionally, the population lacks homogeneity due to different comorbidities, increased competing causes of death, discrepancies in therapy adherence and tolerance, and uneven distribution of cancer stages across groups. The better outcomes found in the surgical population may at least partially be due to selection bias, as surgical patients may present with a more favorable tumor stage. However, the Age-Adjusted Charlson Comorbidity Index was not significantly different among the three treatment groups.

## Conclusions

With the increasing incidence of BC in the oldest old population and the global aging trend, it is imperative to collect stronger evidence on surgical and oncological outcomes for BC patients aged ≥90 years. Rigorous geriatric assessment and a specialized multidisciplinary approach should guide treatment decisions. Our findings underscore the need to tailor the surgical indications for nonagenarians, supporting omission of axillary surgery and highlighting the survival advantages of breast surgery over non-surgical approaches. Consequently, whenever feasible, surgery should be the primary treatment of choice, even within this growing patient demographic, with alternative therapies reserved for patients not suitable for surgery. Aging should not be considered a disease in itself.

## Data Availability

All relevant data are included within this paper and its supporting information files. Anything else required is available upon request.
